# A large outbreak of *Salmonella enterica* serovar Thompson infections associated with chocolate cake in Busan, Korea

**DOI:** 10.4178/epih.e2019002

**Published:** 2019-01-09

**Authors:** Youngduck Eun, Hyesun Jeong, Seungjin Kim, Wonseo Park, Byoungseon Ahn, Dongkeun Kim, Eunhee Kim, Eunhee Park, Sunhee Park, Inyeong Hwang, Hyunjin Son

**Affiliations:** 1 Busan Center for Infectious Disease Control and Prevention, Busan National University Hospital, Busan, Korea; 2 Epidemic Investigation Team of Busan Metropolitan City, Busan, Korea; 3 Division of Health Promotion, Busan Metropolitan City, Busan, Korea; 4 Division of Microbiology, Busan Institute of Health and Environment, Busan, Korea

**Keywords:** Food-borne disease, Salmonella infections, Outbreak, Korea

## Abstract

**OBJECTIVES:**

This study aimed to reveal the epidemiologic characteristics of the outbreak of gastroenteritis caused by *Salmonella enterica* serovar Thompson in Busan Metropolitan City and to identify points for improvement to prevent of food-borne disease outbreak.

**METHODS:**

This was a case-control study. The control group comprised asymptomatic students in the same classes of the cases. The presence or absence of symptoms, ingestion of each food provided by school meal service, and commonly ingested foods in addition to those foods in meal service were investigated. Moreover, specimens collected from rectal swab, preserved foods, and environmental surface were tested.

**RESULTS:**

Of the 6,092 subjects, 1,111 (1,083 students, 22 school personnel, and 6 foodservice employees) were included in the case group; this corresponded to an 18.4% attack rate. Symptoms included diarrhea (n=1,051, 94.6%), abdominal pain (n=931, 83.8%), febrile sensation (n=502, 45.2%), and vomiting (n=275, 24.8%). The epidemic curves of each 10 schools were unimodal. Investigation of food intake showed a significantly high odds ratio for chocolate cake in 5 out of the 10 schools. Laboratory test detected *Salmonella enterica* serovar Thompson both in rectal swab specimens of 9 schools and in collected preserved chocolate cakes of 9 schools. Pulsed-field gel electrophoresis test result showed that *Salmonella enterica* seorvar Thompson isolated from human and foods were the same.

**CONCLUSIONS:**

The source of infection for the *Salmonella enterica* serovar Thompson outbreak in the 10 schools of Busan Metropolitan City is chocolate cake. Traceback investigation for origin of contaminated food in food-borne disease outbreak and safety control during food production should be more enhanced.

## INTRODUCTION

Notified outbreaks of gastroenteritis in South Korea (here after Korea) have continuously increased from 261 cases in 2013 to 422 cases in 2015 and 544 cases in 2017. Of the 544 cases reported in 2017, the most frequent places of outbreak were restaurants (n=260, 47.8%) and schools (n=124, 22.8%). However, the mean case numbers for each outbreak was higher in schools than in restaurants (36 subjects vs. 6 subjects). This shows that students are simultaneously exposed due to mass meal service. Of the 544 cases in 2017, pathogens were identified in 294 (54.0%), which included 101 cases (34.4%) for norovirus, 58 cases (19.7%) for enteropathogenic *Escherichia coli*, 51 cases (17.3%) for *Kudoa septempunctata*, 23 cases (7.8%) for *Salmonella*, and 19 cases (6.5%) for rotavirus [1].

Busan Metropolitan City has received reports from Community Health Centers of each corresponding district, gu, of 4 schools, in that there were several students who complained of gastrointestinal symptoms such as diarrhea, abdominal pain, vomiting, and febrile sensation from 10 a.m. to 3 p.m. in September 5, 2018. Thereafter, additional outbreaks of gastroenteritis were reported from 5 schools on September 6 and 1 school on September 7. On September 6, the Ministry of Food and Drug Safety and the Korea Centers for Disease Control and Prevention (KCDC) announced that 13 schools in the country including Busan reported outbreaks of gastroenteritis until September 5, and the same chocolate cakes provided to those schools were suspected as the source of infection. They then ordered to stop the distribution and sales of this chocolate cake. Thereafter, the Ministry of Food and Drug Safety and the KCDC announced that there were outbreaks of gastroenteritis in 57 mass meal services that provided the chocolate cake in 12 provinces including Busan until September 10, resulting in 2,207 case reports.

This study aimed to reveal the epidemiologic characteristics of the chocolate cake related outbreak in 10 schools of Busan Metropolitan City based on the epidemic investigation results by the Busan Metropolitan City Epidemic Investigation Team and to identify improvement points for prevention and control for and food-borne disease outbreak.

## MATERIALS AND METHODS

### Case definition

Cases were defined based on characteristics of place, person, time, and symptom. Because there were reports on several students who complained of diarrhea, abdominal pain, vomiting, and febrile sensation from September 4, this study defined cases as students, school personnel, or foodservice employees in the 10 schools having one of the following symptoms after September 4: vomiting more than once; diarrhea more than three times in a day; diarrhea more than twice in a day and abdominal pain; and diarrhea more than twice in a day and febrile sensation.

### Epidemic investigation

This study was designed as a case-control study. The control group comprised asymptomatic students in the same classes of case. While the control group was intended to have at least 2 times more students than the case group, 1-3 times more students were recruited depending on the situation of each school. Although if an individual who does not initially belong to either case group or control group develops symptoms that correspond to the case definition during the investigation, they were included in the case group, and if someone in the control group showed symptoms, they were moved to the case group.

The survey included both the case group and the control group as well as foodservice employees. The case group and control group were surveyed for presence or absence of symptoms, number of symptoms, whether they ingested each food provided by each school as meal service from the first day of onset to 2-4 days before, and whether there was any food they commonly had in addition to the meal service. Foodservice employees were surveyed for their participation in cooking for each food in addition to the survey items for case group and control group.

Specimens of rectal swab, preserved foods, environmental surfaces, and water were collected. Rectal swab specimens were collected from some of the subjects in the case group and all 62 foodservice employees in the 10 schools. For preserved foods, a total of 41 meals of the meal service in each of the 10 schools from the onset day to 2-4 days before and 1 box of unopened chocolate cakes were collected and then tested. In addition, 37 environmental surface specimens were collected from cutting boards, knives, and food trays, and 42 water specimens were collected from cooking water and drinking water and then tested.

### Laboratory test

First, the rectal swab specimens were tested for 10 bacterial genus and 5 virus types [1]. After *Salmonella* species was detected, a test for the *Salmonella* species and 5 types of virus was conducted [2].

Regarding the test for preserved foods, the *Salmonella* species similar with that of the rectal swab specimens were detected in the chocolate cake; thus, 28 preserved meals of the days when the corresponding chocolate cake was not provided were excluded from the test, resulting in a test for only 13 preserved meals [3]. Thereafter, preserved food specimens, environmental surface swab specimens, and water specimens were tested for the *Salmonella* genus. For the chocolate cake piece that was preserved, the test was performed for 25 g of each slice without separating bread and cream, and 25 g of cream taken from 1 box of unopened chocolate cake was also tested.

For correlation analysis between the strain isolated from human and the foods, 37 randomly selected strains of each school collected among clinical isolates from rectal swab specimens and all strains isolated from preserved foods were subjected to pulsed-field gel electrophoresis (PFGE) test [4]. PFGE test utilized strains cultured in trypticase soy agar for 20 hours, in which *Xba* I (40U/μL; Roche, Basel, Switzerland) and *Salmonella* serovar Braenderup ATCC BAA-664 were used as restriction enzymes and molecular weight marker, respectively. Electrophoresis was performed at 14°C for 18 hours under the condition of 6.0 V/cm in gradient, 120° in included angle, 2.16 seconds in initial time, and 63.8 seconds in final time, followed by band checking. The results were plotted to a dendrogram in dice similarity index method using BioNumerics (http://www.applied-maths.com/download/software), and then the relationships between strains were analyzed.

### Data analysis

Epidemic curves were created using data of 968 subjects whose time of symptom onset was identified among a total of 1,111 subjects. Odds ratio (OR) and 95% confidence interval (CI) were calculated for ingestion of each food by case group and control group, in which values for unknown or no responses were excluded from analysis. When there was no subject who either did or did not ingest each food in the case group or the control group, 0.5 was added to each cell [5,6].

Data were analyzed using Microsoft Excel 2013 (Microsoft Corp., Redmond, WA, USA) and SAS version 9.4 (SAS Institute Inc., Cary, NC, USA).

## RESULTS

### Attack rate

Of the 6,029 subjects that included students, school personnel, and foodservice employees in the 10 schools, 1,111 met the case definition, corresponding to an 18.4% attack rate (AR). The 1,111 subjects included 1,083 students, 22 school personnel, and 6 foodservice employees. ARs by group were 19.9% for students, 9.7% for foodservice employees, and 4.2% for school personnel. By school, G school showed the highest AR in the 10 schools with 170 (32.9%) out of 516 subjects, while E school showed the lowest AR with 15 (5.0%) out of 302 subjects (Table 1). Cases were relatively evenly distributed throughout all grades of each 10 school.

The most common symptoms of the 1,111 cases were diarrhea (n=1,051, 94.6%), abdominal pain (n=931, 83.8%), febrile sensation (n=502, 45.2%), and vomiting (n=275, 24.8%) (Table 2).

Of the 1,111 subjects, 151 subjects (13.6%) including 149 students and 2 school personnel were hospitalized. The mean hospitalization duration was 5 days (range, 2.3 to 7.7). For E school, 11 (73.3%) of the 15 subjects were hospitalized, corresponding to the highest hospitalization rate, and they also had the longest mean hospitalization duration of 7.7 days (Table 3).

### Epidemic curve

**Figure 1.**
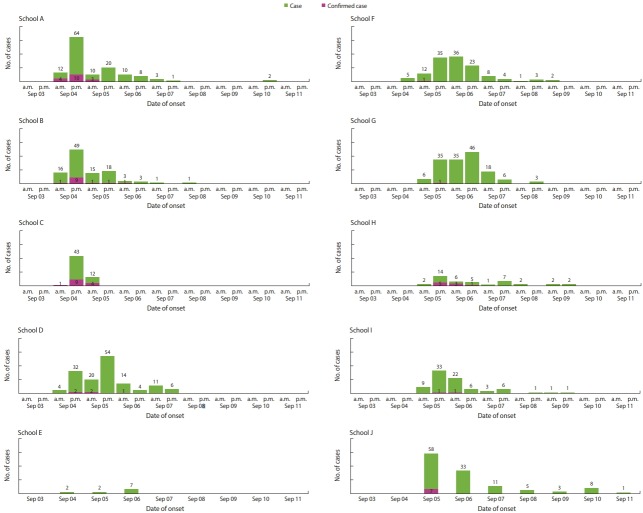
Epidemic curves of each 10 affected schools.

The epidemic curves of 8 of the 10 schools (except E and H schools, which had the least number of cases) were unimodal, suggesting outbreak from single common exposure (Figure 1). Peaks of the epidemic curve appeared on the first day of outbreak in A, B, C, H, I, and J schools and the second day of outbreak in D, F, and G schools, of which A and J schools were reported to have cases until 6 days even after reaching the peak of epidemic curve.

### Odds ratio of ingestion by meal service food in the case-control group

Of the 10 schools, 6 schools had foods with significant OR and 95% CI for ingestion. Of them, A, F, G, I, and J schools showed a significant OR for chocolate cake that was provided by a specific company in a complete product form. In particular, G and I schools had as high as 39.4 (95% CI, 5.4 to 289.5) and 30.3 (95% CI, 1.8 to 518.8) OR for chocolate cake, respectively (Table 4).

### Laboratory test results

Rectal swab specimens were collected and tested from 205 subjects in the case group. *Salmonella* Thompson was detected in 89 subjects (43.8%) of 9 schools except E school. For E school, rectal swab specimens were collected from 3 cases, and *Salmonella* Thompson was not detected (Table 5). Of the 89 subjects whose rectal swab specimens were found to have *Salmonella* Thompson, 2 subjects were foodservice employees, and they reported that they consumed the same meal with students.

When preserved foods were tested, *Salmonella* Thompson was detected from all chocolate cakes collected from 9 out of the 10 schools. Of the 11 cases of preserved foods provided on the day when chocolate cake was provided in each school, *Salmonella* species was not detected in foods except chocolate cake. When only the cream part from 1 box of unopened chocolate cake among the preserved foods in F school was tested, *Salmonella* Thompson was detected.

No *Salmonella* species was detected in 37 cases of environmental surface swab specimens and 42 cases of water specimens including cooking water and drinking water.

We were conducted PFGE tests for clinical isolates and 9 isolates form chocolate cake of each school. All 46 isolates had patterns with 100% similarity; thus, it was confirmed that the *Salmonella* Thompson isolated from human and foods were the same.

### Control measures

Hygiene education, environmental disinfection, and active surveillance on additional case were implemented. Such measures were continued until the end of the outbreak. Handwashing was emphasized in hygiene education, which was targeted to all students and staff in the schools. Community Health Centers provided schools with hand sanitizers. For environmental disinfection, the surfaces of school restrooms and door knobs were disinfected using sodium hypochlorite at least once a day. Community Health Centers received daily reports on the situation of active surveillance on additional case and hospitalization. In addition, schools sent newsletters and text messages to inform parents of the situation of outbreak and precautions. Until the end of outbreak, only cooked foods and boiled water were provided in meal services. Foodservice employees who either had symptoms or were detected to have the pathogen were suspended from cooking and were tested 2 times every 24 hours after 48 hours from taking antibiotics. They were allowed to return to cooking after negative results were confirmed. Schools with persisting cases were instructed to recheck handwashing methods and environmental disinfection, and their school staff in charge of control were requested to take appropriate measures for epidemic control.

## DISCUSSION

There are over 2,500 serovars of the Salmonella genus [7]. Of them, only *Salmonella* Typhi and *Salmonella* Paratyphi have humans as the host, causing enteric fever. Meanwhile, the remaining serovars are classified as non-typhoidal *Salmonella*, which can form colonies in gastrointestinal tracts of various animals including mammals, reptiles, birds, and insects. Of the non-typhoidal *Salmonella*, over 200 serovars have pathogenicity to humans, frequently causing gastroenteritis and local infection and bacteremia [8]. Non-typhoidal *Salmonella* is the major causative pathogen of bacterial diarrhea and is also believed to cause gastroenteritis in 150 million individuals and 57,000 deaths every year [9].

The incubation period of gastroenteritis caused by infection of non-typhoidal *Salmonella* is roughly 6-72 hours and 12-36 hours on average. The most common symptoms include acute diarrhea, abdominal pain, fever, and vomiting, which last for 4-7 days and resolve without treatment in most cases [10]. However, infants, the elderly, and those who are immune compromised may require hospitalization and antibiotic treatment due to the potential for dehydration and disseminated infection [8].

Because non-typhoidal Samonella forms colonies in gastrointestinal tracts of various animals, it is often detected in cases in which humans were infected by eating contaminated agricultural, fishery, and livestock products including poultry meat, egg, minced beef, milk, vegetables, and fruits [11-18]. In addition, there were reports on transmission through various processed foods including chocolate bar, peanut butter, orange juice, and smoked salmon [19-22].

Through the epidemic investigation on the simultaneous outbreak of gastroenteritis in the 10 schools of Busan Metropolitan City, it was confirmed that the pathogen was *Salmonella* Thompson and the source of infection was chocolate cake. Most schools showed unimodal epidemic curves, indicating that the outbreak was attributable to single common exposure. The symptoms of the case group were consistent with gastroenteritis caused by non-typhoidal *Salmonella*, in which diarrhea and fever are the primary symptoms. Cases were evenly distributed through all grades that had meal service in each school. Regarding OR for ingestion of chocolate cake from the same company, 5 schools commonly showed significant values, of which 2 schools had OR as high as 39.4 (95% CI, 5.4 to 289.5) and 30.3 (95% CI, 1.8 to 518.8). *Salmonella* Thompson was detected in rectal swab specimens or the chocolate cake in all 10 schools, and all PFGE types were consistent.

**Figure 2.**
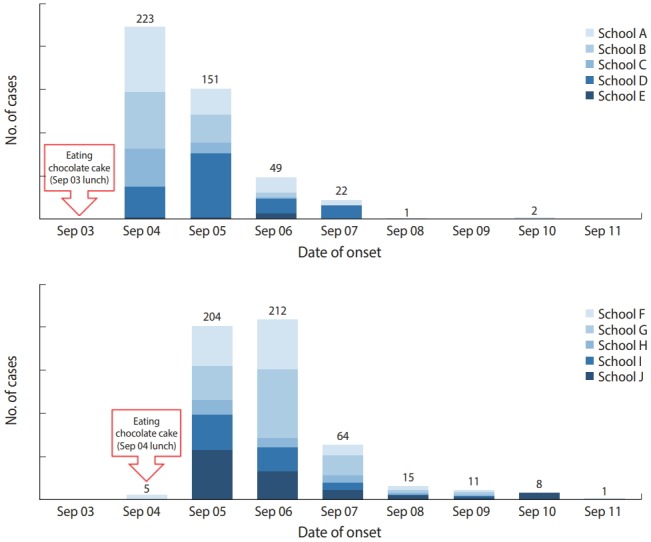
Epidemic curves of each 5 schools by chocolate cake intake date.

Chocolate cakes were provided during lunch in 5 of the 10 schools on September 3 and at the remaining 5 schools on September 4. Epidemic curves by date when chocolate cake was provided showed that symptoms occurred between 6 hours and 72 hours, which corresponded to the incubation period of non-typhoidal *Salmonella* (Figure 2). Some schools showed epidemic curves with either long tail or rebound in the case number, which can be speculated that either some cases among those caused by single common exposure had long incubation period, or the other cases had secondary small-scale infections due to person to person transmission [9,23].

Because F school stored a sufficient amount of the packed chocolate cake, the cream in the cake was tested separately, and *Salmonella* Thompson was detected in the cream. Thereafter, the Ministry of Food and Drug Safety announced that the egg white used for making the cream was infected with *Salmonella* Thompson and was the source of infection for the chocolate cake.

Lee et al. [24] pointed that no study reported detection of *Salmonella* in eggs, although there were reports on outbreaks of non-typhoidal salmonellosis which was related with egg or foods made of egg in Korea. Thus, a nationwide investigation to produce reliable data is needed. To prevent the outbreak of food-borne infection, an integrated and systematic surveillance system that covers raw materials of agricultural, fishery, and livestock products as well as gastroenteritis pathogens of humans should be established. In addition, there is traceback investigation for origin of contaminated food when there are local outbreaks of food-borne disease. Thus, more thorough traceback investigation should be performed to prevent such nationwide large-scale outbreaks.

This large-scale epidemic outbreak in Busan occurred simultaneously in the 10 schools, and it was primarily caused by the cakes provided in the beginning of month to celebrate students’ birthdays. Of the schools in Busan Metropolitan City, 30 schools served the chocolate cakes on September 3 (Monday) and September 4 (Tuesday), of which only 10 schools that provided chocolate cakes with expiration dates of April 9, 10, or 11 in 2019 had outbreak. Thus, it could be speculated that chocolate cakes produced on specific dates were contaminated. For foods supplied to mass meal services in the form of ready-to-eat products, contamination in the production stage could cause a large-scale epidemic outbreak. Thus, safety control during production should be enhanced. Because the major cause of the large-scale outbreak was a ready-to-eat product that is simultaneously supplied to schools nationwide, institutions using large-scale mass meal services such as schools should take measures to prevent simultaneous exposing students to contaminated food.

This study has some limitations. First, a retrospective cohort investigation that included all students in the school was not performed. In addition, the specific time of symptom onset for 143 subjects (12.8%) in the case group was not investigated; thus, the epidemic curve was incomplete. For a retrospective cohort investigation that includes all students in the school, we need to request the students to remain in the school even after class hours, which is practically difficult. Thus, we could only perform a case-control study. If an online survey method was developed instead of the currently used paper questionnaire, it would be feasible to apply a retrospective cohort design. Due to the reports of simultaneous outbreak in the 10 schools within 3 days, the investigation was performed focusing on rapidity, not on completeness. Although there were some missing items in survey responses, the survey and the tests on human body specimens, preserved foods, and environmental specimens were performed completely in all 10 schools.

The “Busan Metropolitan City water-borne and food-borne disease outbreak working group” which was established in 2017, played a significant role in the rapid and smooth conduct of the large-scale epidemic investigation in Busan Metropolitan City. This was because a system had been already established for epidemic investigations in cases of infectious disease outbreaks in schools and for sharing data. The working group is composed of the Busan Metropolitan City Epidemic Investigation Team, Busan Institute of Health and Environment, Community Health Centers of gu/gun, Food Sanitation Divisions, the Education Offices, and Busan Regional Office of Food and Drug Safety. A horizontal, voluntary, and collaborative relationship among institutions or divisions such as in this working group is a highly important factor for the smooth execution of epidemic investigations of infectious diseases.

## Figures and Tables

**Table 1.** Attack rate in the 10 affected schools by subject

School	Total	Cases	Student		School personnel		Foodservice employee	
			Total	Cases	Total	Cases	Total	Cases
A	679	147 (21.6)	599	139 (23.2)	73	8 (11.0)	7	0 (0.0)
B	710	127 (17.9)	650	121 (18.6)	53	3 (5.7)	7	3 (42.9)
C	484	93 (19.2)	429	91 (21.2)	50	2 (4.0)	5	0 (0.0)
D	484	145 (30.0)	424	138 (32.5)	52	7 (13.5)	8	0 (0.0)
E	302	15 (5.0)	267	15 (5.6)	31	0 (0.0)	4	0 (0.0)
F	788	157 (19.9)	718	157 (21.9)	63	0 (0.0)	7	0 (0.0)
G	516	170 (32.9)	457	167 (36.5)	53	2 (3.8)	6	1 (16.7)
H	837	45 (5.4)	778	45 (5.8)	52	0 (0.0)	7	0 (0.0)
I	289	86 (29.8)	242	86 (35.5)	43	0 (0.0)	4	0 (0.0)
J	940	126 (13.4)	880	124 (14.1)	53	0 (0.0)	7	2 (28.6)
Total	6,029	1,111(18.4)	5,444	1,083 (19.9)	523	22 (4.2)	62	6 (9.7)

Values are presented as number or number (%).

**Table 2.** Symptom distribution in the 10 affected schools

School	No. of cases	Symptom^1^				
		Diarrhea	Abdominal pain	Febrile sensation	Vomiting	Headache
A	147	141 (95.9)	132 (89.8)	52 (35.4)	31 (21.1)	14 (9.5)
B	127	120 (94.5)	117 (92.1)	88 (69.3)	40 (31.5)	0 (0.0)
C	93	86 (92.5)	44 (47.3)	19 (20.4)	52 (55.9)	7 (7.5)
D	145	145 (100)	135 (93.1)	47 (32.4)	17 (11.7)	0 (0.0)
E	15	14 (93.3)	3 (20.0)	4 (26.7)	10 (66.7)	0 (0.0)
F	157	153 (97.5)	127 (80.9)	83 (52.9)	24 (15.3)	0 (0.0)
G	170	163 (95.9)	161 (94.7)	71 (41.8)	42 (24.7)	0 (0.0)
H	45	42 (93.3)	41 (91.1)	25 (55.6)	8 (17.8)	0 (0.0)
I	86	86 (100)	67 (77.9)	33 (38.4)	7 (8.1)	0 (0.0)
J	126	101 (80.2)	104 (82.5)	80 (63.5)	44 (34.9)	0 (0.0)
Total	1,111	1,051 (94.6)	931 (83.8)	502 (45.2)	275 (24.8)	21 (1.9)

Values are presented as number (%).

1 Respondents could select multiple choices.

**Table 3.** Hospitalization rate in the 10 affected schools

School	No. of cases	Hospitalized cases	Student	School personnel	Mean duration of hospitalization (d)
A	147	31 (21.1)	30	1	6.2
B	127	7 (5.5)	7	0	4.9
C	93	27 (29.0)	27	0	4.3
D	145	23 (15.9)	23	0	6.6
E	15	11 (73.3)	11	0	7.7
F	157	7 (4.5)	7	0	2.3
G	170	23 (13.5)	22	1	6.0
H	45	8 (17.8)	8	0	4.0
I	86	5 (5.8)	5	0	4.2
J	126	9 (7.1)	9	0	3.7
Total	1,111	151 (13.6)	149	2	5.0

Values are presented as number or number (%).

**Table 4.** ORs for foods with significant results by school

School	Food	Date of ingestion	Case		Control		OR (95% CI)^1^
			Ingestion	Non-ingestion	Ingestion	Non-ingestion	
A	Seasoned mung bean sprouts	03-Sep (lunch)	46 (70.8)	19 (29.2)	67 (54.5)	56 (45.5)	2.0 (1.1, 3.8)
	Chocolate cake	03-Sep (lunch)	63 (95.5)	3 (4.5)	106 (84.1)	20 (15.9)	4.0 (1.1, 13.9)
B	Mushroom soybean paste stew	31-Aug (lunch)	30 (37.5)	50 (62.5)	43 (24.7)	131 (75.3)	1.8 (1.0, 3.2)
	Vegetable side dishes	03-Sep (lunch)	46 (58.2)	33 (41.8)	76 (42.5)	103 (57.5)	1.9 (1.1, 3.2)
F	Diced radish kimchi	03-Sep (lunch)	53 (63.9)	30 (36.1)	107 (43.5)	139 (56.5)	2.3 (1.4, 3.8)
	Diced radish kimchi	03-Sep (dinner)	25 (30.1)	58 (69.9)	47 (19.1)	199 (80.9)	1.8 (1.0, 3.2)
	Rice with bean	04-Sep (lunch)	81 (97.6)	2 (2.4)	219 (89.0)	27 (11.0)	5.0 (1.2, 21.5)
	Abalone seaweed soup	04-Sep (lunch)	70 (84.3)	13 (15.7)	173 (70.3)	73 (29.7)	2.3 (1.2, 4.4)
	Braised pork and ripe kimchi	04-Sep (lunch)	81 (97.6)	2 (2.4)	195 (79.3)	51 (20.7)	10.6 (2.5, 44.5)
	Squid chive pancake	04-Sep (lunch)	69 (83.1)	14 (16.9)	165 (67.1)	81 (32.9)	2.4 (1.3, 4.6)
	Sesame leaves in soy sauce	04-Sep (lunch)	65 (78.3)	18 (21.7)	157 (63.8)	89 (36.2)	2.0 (1.1, 3.7)
	Chocolate cake	04-Sep (lunch)	92 (97.9)	2 (2.1)	207 (84.1)	39 (15.9)	8.7 (2.0, 36.7)
G	Brown rice and boiled glutinous rice	04-Sep (lunch)	86 (96.6)	3 (3.4)	153 (78.9)	41 (21.1)	7.7 (2.3, 25.5)
	Stir-fried octopus	04-Sep (lunch)	82 (92.1)	7 (7.9)	149 (76.8)	45 (23.2)	3.5 (1.5, 8.2)
	Vegetables rolled omelet	04-Sep (lunch)	85 (95.5)	4 (4.5)	148 (77.5)	43 (22.5)	6.2 (2.1, 17.8)
	Chocolate cake	04-Sep (lunch)	88 (98.9)	1 (1.1)	134 (69.1)	60 (30.9)	39.4 (5.4, 289.5)
I	Red bean rice	04-Sep (lunch)	67 (98.5)	1 (1.5)	70 (88.6)	9 (11.4)	8.6 (1.1, 69.9)
	Beef and seaweed soup	04-Sep (lunch)	63 (92.6)	5 (7.4)	59 (76.6)	18 (23.4)	3.8 (1.3, 11.0)
	Andong braised chicken	04-Sep (lunch)	68 (100)	0 (0.0)	69 (87.3)	10 (12.7)	20.6 (1.2, 360.2)
	Chocolate cake	04-Sep (lunch)	68 (100)	0 (0.0)	65 (82.3)	14 (17.7)	30.3 (1.8, 518.8)
J	Chocolate cake	04-Sep (lunch)	90 (96.8)	3 (3.2)	371 (88.3)	49 (11.7)	4.0 (1.2, 13.0)

Values are presented as number (%). OR, odds ratio; CI, confidence interval.

1 In case the counts of each cell contains zero, the OR and 95% CI were calculated by adding 0.5 to each cell.

**Table 5.** Laboratory test result of rectal swab specimens in the 10 affected schools

School	Total (n)^a^	Tested cases		Confirmed cases^1^	
		n^b^	b/a (%)	n^c^	c/b (%)
A	147	34	23.1	20	58.8
B	127	35	27.6	15	42.9
C	93	55	59.1	23	41.8
D	145	6	4.1	5	83.3
E	15	3	20.0	0	0.0
F	157	23	14.6	7	30.4
G	170	9	5.3	1	11.1
H	45	21	46.7	9	42.9
I	86	6	7.0	2	33.3
J	126	13	10.3	7	53.8
Total	1,111	205	18.5	89	43.4

1 *Salmonella enterica* serovar Thompson was detected in all confirmed cases.

## SUPPLEMENTARY MATERIALS

Korean version is available at http://www.e-epih.org/).
