# Supplementary material for: A large outbreak of Salmonella enterica serovar Thompson infections associated with chocolate cake in Busan, Korea
Source: Epidemiol Health. 2019 Jan 9;41:e2019002. doi: 10.4178/epih.e2019002 (PMC6391592; doi:10.4178/epih.e2019002)
Supplement: Supplementary file 1 [file epih-41-e2019002-supplementary.pdf]

Volume: 41, Article ID: e2019002
https://doi.org/10.4178/epih.e2019002

A large outbreak of *Salmonella enterica* serovar Thompson Infections Associated with Chocolate Cake in Busan

부산광역시 초콜릿 케이크 관련 살모넬라 톰슨 감염증 대규모 집단발생

저자: 은영덕[1)2)], 정혜선[1)2)], 김승진[1)2)], 박원서[1)], 안병선[2)3)], 김동근[2)3)], 김은희[2)3)], 박은희[2)4)], 박선희[4)], 황인영[4)], 손현진[1)2)]

소속: 1) 부산대학교병원 부산광역시 감염병관리지원단 2) 부산광역시 역학조사반 3) 부산광역시 건강증진과 4) 부산광역시 보건환경연구원 미생물검사팀

저자(영문): Youngduck Eun[1)2)], Hyesun Jeong[1)2)], Seungjin Kim[1)2)], Wonseo Park[1)], Byoungseon Ahn[2)3)], Dongkeun Kim[2)3)], Eunhee Kim[2)3)], Eunhee Park[2)4)], Sunhee Park[4)], Inyeong Hwang[4)], Hyunjin Son[1)2)]

소속(영문): 1) Busan Center for infectious Disease Control and Prevention, Busan National University Hospital, Busan, Korea 2) Epidemic investigation team of Busan Metropolitan City, Busan, Korea 3) Division of Health Promotion, Busan Metropolitan City, Busan, Korea 4) Division of Microbiology, Busan Institute of Health and Environment, Busan, Korea

책임저자: 손현진(Hyunjin Son), 부산광역시 연제구 중앙대로 1001 부산광역시청 14층(14F, 1001 Jungang-daero, Yeonje-gu, Busan, Korea 47545), hjson78@gmail.com

초록 Abstract

목적(Objectives): 2018년 9월 부산광역시 소재 10개 학교에서 살모넬라 톰슨에 의한 장관감염증이 집단발생하였다. 이 연구는 부산광역시 역학조사반의 역학조사 결과를 바탕으로 유행의 역학적 특성과 원인을 밝히고 향후 식품매개 감염병 집단발생의 예방과 유행관리에 대한 개선점을 도출하고자 하였다.

방법(Methods): 사례-대조군 연구를 수행하였다. 대조군은 사례와 같은 반 학생 중 증상이 없는 경우로 하였다. 증상 여부, 학교 급식으로 제공된 식품별 섭취여부, 급식 외 공동으로 먹은 음식에 대한 설문조사를 실시하였고, 직장도말, 보존식, 환경표면 검체를 채취하여 검사하였다.

결과(Results): 부산광역시 소재 10개 학교의 학생, 교직원, 조리종사자 총 6,092명 중 1,111명이 사례 정의에 부합하여 발병률은 18.4%였다. 사례군 중 학생은 1,083명, 교직원은 22명, 조리종사자는 6명이었다. 증상은 설사 94.6% (1,051명), 복통 83.8% (931명), 발열감 45.2% (502명), 구토 24.8% (275명) 순이었으며 사례군 중 151명(13.6%)이 입원하였다. 각 학교의 유행곡선은 단봉형으로 단일 공동노출에 의한 유행 양상을 보였다. 식품섭취력 조사 결과 10개 학교 중 5개 학교에서 초콜릿 케이크의 오즈비가 의미있게 높았다. 특히 한 학교에서는 초콜릿 케이크의 오즈비가 39.4(95% CI 5.4-289.5)로 매우 높았다. 실험실 검사 결과 9개 학교의 직장도말 검체에서 살모넬라 톰슨이 검출되었으며, 수거된 보존식 중 9개 학교의 초콜릿 케이크에서 살모넬라 톰슨이 검출되었다. 직장도말 검체와 초콜릿 케이크에서 검출된 균주의 유전자 지문분석(PFGE) 검사로 10개교의 인체와 식품에서 검출된 원인병원체가 동일함을 확인하였다.

결론(Conclusions): 부산광역시 10개 학교에서 발생한 살모넬라 톰슨 집단발생의 감염원은 초콜릿 케이크로 확정할 수 있다. 식품매개 감염병 집단발생 원인 식품의 원재료에 대한 역추적 조사가 보다 철저히 이루어져야 하며, 식품 생산 단계에서의 안전관리가 더욱 강조되어야 한다.

* 중심단어(Key words) - Food borne disease, Salmonella infections, Outbreak, Korea

서론 Introduction

우리나라에서 보고된 수인성·식품매개 감염병 집단발생은 2013년 261건에서 2015년 422건, 2017년 544건으로 지속적으로 증가하고 있다. 2017년 보고된 544건을 발생 장소별로 살펴보면 식당에서 발생한 경우가 260건(47.8%)으로 가장 많았고 학교가 124건(22.8%)으로 두 번째로 많았다. 그러나 집단발생 건당 평균 사례 수는 식당은 6명, 학교는 36명으로 나타나고 있어 학교의 경우 집단급식으로 한 학교의 모든 학생이 동시에 노출되는 특성을 반영하고 있다. 2017년 발생한 544건 중 294건(54.0%)에서 원인병원체가 규명되었는데, 노로바이러스 101건(34.4%), 병원성대장균 58건(19.7%), 쿠도아충 51건(17.3%), 살모넬라 23건(7.8%), 로타바이러스 19건(6.5%)의 순이었다[1].

2018년 9월 5일 오전 10시부터 오후 3시까지 부산시 3개 구 4개 학교에서 설사와 복통, 구토, 발열 등 장관감염 증상을 호소하는 학생이 다수 발생하였다는 신고가 각 구 보건소를 통해 부산광역시로 보고된 것을 시작으로, 다음 날인 9월 6일에는 5개 학교, 9월 7일 1개 학교에서 추가로 장관감염증 집단발생이 보고되었다. 9월 6일 식품의약품안전처와 질병관리본부는 9월 5일까지 부산을 비롯해 전국 13개 학교에서 장관감염증 집단발생이 보고되었으며 이들 학교에 동일하게 공급된 초콜릿 케이크가 감염원으로 추정된다고 발표하고 해당 초콜릿 케이크의 유통과 판매를 중단하도록 조치하였다. 이후 식품의약품안전처와 질병관리본부는 9월 10일까지 부산을 포함한 총 12개 광역자치단체에서 해당 초콜릿 케이크가 급식으로 제공된 집단급식소 중 57개에서 장관감염증이 집단발생 하였고 보고된 사례는 2,207명에 이른다고 발표하였다.

이 연구에서는 부산광역시 역학조사반이 조사한 부산광역시 소재 10개 학교의 초콜릿 케이크 관련 장관감염증 집단발생 역학조사 결과를 바탕으로 유행의 역학적 특성과 원인을 밝히고 향후 수인성·식품매개 감염병 집단발생 예방과 유행관리에 대한 개선점을 도출하고자 하였다.

대상 및 방법 Materials and Methods

사례 정의 Case definition

사례는 장소, 사람, 시간, 증상의 특성으로 정의하였다. 9월 4일부터 설사, 복통, 구토, 발열감을 호소하는 학생이 다수 보고되고 있었으므로, 사례 정의는 10개 학교의 학생과 교직원, 조리종사자 중 9월 4일 이후 다음 중 하나 이상에 해당하는 증상이 있는 경우로 하였다.

- 구토 1회 이상이 있는 경우
- 하루에 설사 3회 이상이 있는 경우
- 하루에 설사 2회 이상과 복통이 있는 경우
- 하루에 설사 2회 이상과 발열감이 있는 경우

역학조사 내용 Epidemic investigation

조사디자인은 사례-대조군 연구로 하였다. 대조군은 사례와 같은 반 학생 중 증상이 없는 경우로 하였다. 대조군은 사례의 2배수 이상 선정하도록 하였으나 각 학교별 상황에 따라 사례군의 1-3배로 선정되었다. 조사 초기에 사례군이나 대조군에 속하지 않았더라도 조사 진행 과정 중 증상이 발현되어 사례정의에 부합한 경우 사례군에 포함하였고, 대조군 중 증상이 발현된 경우는 사례군으로 변경하였다.

설문조사는 사례군과 대조군, 조리종사자를 대상으로 하였다. 사례군과 대조군을 대상으로 한 설문 내용은 증상 발생 여부와 횟수, 각 학교별 최초 증상 발생일부터 2-4일 이전까지 학교에서 급식으로 제공한 식품별 섭취 여부, 급식 외 공동으로 섭취한 음식에 대한 것이었다. 조리종사자에게는 사례군과 대조군 대상 설문 내용에 식품별 조리 참여 여부도 추가하여 조사하였다.

직장도말, 보존식, 환경표면, 물 검체를 채취하였다. 직장도말 검체는 사례군 일부와 10개 학교 조리종사자 62명 전원을 대상으로 채취하였다. 보존식은 10개 학교별 최초 증상 발생일부터 2-4일 이전까지의 급식 총 41식과 개봉하지 않은 초콜릿 케이크 1 박스를 수거하여 검사하였다. 환경표면 검체는 도마·칼·식판 등 37건, 물은 조리수·정수기 음용수 등 42건을 채취하여 검사하였다.

실험실 검사 Laboratory test

직장도말 검체에 대해 초기에는 세균 10균속 및 바이러스 5종 검사를 실시하였으나[1], 살모넬라균이 검출된 이후부터는 살모넬라균속과 바이러스 5종에 대하여 검사를 실시하였다[2].

보존식에 대한 검사 진행 초기에 조각 초콜릿 케이크에서 직장도말 검체와 같은 살모넬라균이 검출되어 해당 초콜릿 케이크가 제공되지 않은 날의 보존식 28식은 검사에서 제외하였고 총 13식의 보존식에 대한 검사가 이루어졌다[3]. 이후 보존식과 환경표면 도말검체, 물 검체에 대해 살모넬라균속 검사를 시행하였다. 보존식인 조각 초콜릿 케이크는 빵과 크림 구분 없이 25g씩 검사하였으며 개봉하지 않은 채 수거된 초콜릿 케이크 1 박스에서는 크림 부분만 25g을 취하여 검사하였다.

인체에서 분리된 균주와 식품에서 분리된 균주 간 유연관계 분석을 위해 직장도말 검체에서 분리된 인체 균주 중 학교별로 무작위 선정한 37주와 보존식에서 분리된 모든 균주에 대해 다방향 전기영동(PFGE, Pulsed Field Gel Electrophoresis) 검사를 실시하였다[4]. PFGE 검사는 대두카제인소화한천배지(TSA, Trypticase Soy Agar)에 20시간 배양된 균주를 사용하였으며, 제한효소는 Xba I (Roche, 40U/㎕, Basel, Switzerland)를, molecular weight marker는 *Salmonella* serovar Braenderup ATCC BAA-664를 사용하였다. gradient 6.0 V/cm, included angle 120°, initial time 2.16초, final time 63.8초의 조건으로 14℃에서 18시간 전기영동을 실시하여 밴드를 확인하였으며 이 결과는 BioNumerics software(Applied Maths, Belgium)를 이용하여 Dice similarity index 방법으로 Dendrogram을 작성하여 균주간의 유연관계를 분석하였다.

자료 분석 Data analysis

유행곡선은 총 사례 1,111명 중 증상이 시작된 날짜 또는 시각이 확인된 968명의 자료를 이용하여 작성하였다. 사례군과 대조군의 식품별 섭취 여부에 대한 오즈비와 95% 신뢰구간을 구하였으며 모름 또는 무응답 값은 분석에서 제외하였다. 사례군과 대조군에서 식품별 섭취 또는 비섭취 군이 0 명인 경우는 각각의 빈도에 0.5를 추가하여 계산하였다[5,6].

자료는 Microsoft Excel 2013 (Microsoft Corp., Redmond, WA, USA) 및 SAS software, version 9.4 (SAS Institute, Cary, North Carolina, USA)을 이용하여 분석하였다.

결과 Results

발병률 Attack rate

10개 학교의 학생, 교직원, 조리종사자 총 6,029명 중 1,111명이 사례 정의에 부합하여 발병률은 18.4%였다. 사례 1,111명 중 학생은 1,083명, 교직원은 22명, 조리종사자는 6명이었으며, 집단별 발병률은 학생 19.9%, 조리종사자 9.7%, 교직원 4.2% 였다. 학교별 발병률을 보면 10개 학교 중 가장 높은 발병률을 보인 곳은 G학교로 516명 중 170명(32.9%)이 사례로 분류되었으며, 가장 낮은 발병률을 보인 곳은 E학교로 302명 중 15명(5.0%)이 사례로 분류되었다 (Table 1). 10개 학교 모두 전 학년에서 사례가 비교적 고르게 발생하였음을 확인하였다.

사례 1,111명의 증상은 설사 94.6% (1,051명), 복통 83.8% (931명), 발열감 45.2% (502명), 구토 24.8% (275명) 순으로 많았다 (Table 2).

입원 치료를 받은 사례는 1,111명 중 151명(13.6%)으로, 학생이 149명, 교직원이 2명이었다. 평균 입원일수는 5일로 학교별 평균 입원일수는 2.3-7.7일이었다. E학교의 경우 15명 중 11명

(73.3%)이 입원하여 가장 높은 입원율을 보였고 평균 입원일수도 7.7일로 가장 길었다 (Table 3).

유행 곡선 Epidemic curve

10개 학교 중 사례 수가 적었던 E, H 학교를 제외한 8개 학교에서 단봉형 유행곡선을 보여 단일 공동노출에 의한 유행의 가능성을 시사하였다 (Figure 1). A, B, C, H, I, J 학교는 유행 첫날에 D, F, G 학교는 유행 둘째 날에 유행곡선의 정점을 보였으며, A, J 학교는 유행 곡선의 정점에서 6일 이후까지 사례가 보고되었다.

사례-대조군 급식 식품별 섭취 여부 오즈비

식품섭취 여부에 대한 오즈비와 95% 신뢰구간 분석 결과 유의미한 값을 보인 식품이 있었던 학교는 10개 학교 중 6개 학교였다. 이 중 A, F, G, I, J 5개 학교에서 동일 회사에서 완제품 형태로 공급한 조각 초콜릿 케이크의 오즈비가 공통적으로 의미있는 값을 보였다. 특히 G학교와 I학교의 경우 초콜릿 케이크의 오즈비가 각각 39.4(95% CI 5.4-289.5), 30.3(95% CI 1.8-518.8)으로 매우 높았다 (Table4).

실험실 검사 결과

사례군 205명에서 직장도말 검체를 채취하여 검사한 결과 E학교를 제외한 9개 학교의 사례 89명(43.8%)에서 살모넬라 톰슨이 검출되었다. E학교의 경우 사례 3명에서 직장도말 검체가 채취되었으나 살모넬라 톰슨은 검출되지 않았다 (Table 5). 직장도말 검체에서 살모넬라 톰슨이 검출된 89명 중 2명은 조리종사자였으며 이들은 학생들과 동일한 급식을 섭취했다고 응답하였다.

보존식 검사 결과 10개 학교 중 G학교를 제외한 9개 학교에서 수거된 초콜릿 케이크에서 모두 살모넬라 톰슨이 검출되었다. 각 학교에서 초콜릿 케이크가 제공된 날의 보존식 11건 중 초콜릿 케이크를 제외한 식품에서는 살모넬라균이 검출되지 않았다. F학교에서 보관 중이었던 개봉하지 않은 초콜릿 케이크 1박스에서 크림 부분만을 검사한 결과 살모넬라 톰슨이 검출되었다.

환경표면 도말검체 37건과 조리수·정수기 음용수 등 42건의 물 검체에서는 살모넬라균속이 검출되지 않았다.

학교별로 무작위 선정된 인체 검출 살모넬라 톰슨 37주와 초콜릿 케이크에서 검출된 살모넬라 톰슨 9주에 대한 유전자 지문분석(PFGE) 검사 결과 모두 동일한 유형으로 나타나 인체와 식품에서 분리된 살모넬라 톰슨은 동일한 균주임을 확인할 수 있었다.

관리 조치 Control measures

위생 교육과 환경 소독, 추가 사례 능동감시를 시행하였다. 이러한 조치들은 유행이 종료 될 때까지 지속하도록 하였다. 위생 교육을 통해 손 씻기 실천을 강조하였으며 학교 내 학생과 교직원 전체를 대상으로 하였다. 보건소에서는 각 학교에 손소독제를 제공하였다. 환경 소독은 학교 화장실과 문손잡이 등 학생들이 주로 접촉하는 표면을 염소계 소독제를 이용하여 하루 한번 이상 소독하도록 하였다. 추가 사례 능동 감시 결과와 입원 현황을 학교에서 보건소로 매일 보고하도록 하였다. 또한 학교에서는 가정통신문과 휴대전화 메세지를 통해 학부모에게 유행 발생 상황과 주의사항을 안내하였다. 유행종료 시까지 익힌 음식과 끓인 물만을 급식으로 제공하도록 하였다. 조리종사자 중 증상이 있거나 원인병원체가 검출된 경우 조리 업무를 중단하도록 하였으며, 항생제 복용 종료 48시간 후 24시간 간격으로 2회 검사를 시행하고 음성결과를 확인한 후 조리 업무에 복귀하도록 하였다. 사례 발생이 지속되는 학교는 손 씻기와 환경소독에 대해 재점검하고 학교 관리자를 대상으로 유행 관리 조치가 잘 이루어지도록 당부하였다.

고찰 Discussion

살모넬라속 세균에는 2,500가지 이상의 혈청형이 존재한다[7]. 그 중 *Salmonella* Typhi 와 *Salmonella* Paratyphi 는 사람이 유일한 숙주로 창자열(enteric fever)의 원인이 되며, 나머지 혈청형은 non-typhoidal *Salmonella*로 분류되고 포유류, 파충류, 조류, 곤충을 포함하는 다양한 동물들의 위장관내에 집락을 형성할 수 있다. 이들 non-typhoidal *Salmonella* 중 200여종 이상의 혈청형이 사람에게 병원성을 보여 위장관염을 흔히 일으키고 국소감염과 세균혈증 등을 일으킬 수 있다[8]. Non-typhoidal *Salmonella*는 전세계적으로 세균성 설사의 주요 원인균이며 전세계에서 매년 1억 5천만 명에게 위장관염을 일으키고 5만 7천명의 사망자를 내는 것으로 추정된다[9].

Non-typhoidal *Salmonella* 감염으로 인한 위장관염의 잠복기는 넓게는 6-72시간이며 보통 12-36 시간이다. 주로 급성 설사, 복통, 발열, 구토의 증상을 나타내며 증상은 4-7일간 계속되고 대부분은 치료 없이 회복되지만[10], 신생아, 노인, 면역저하자는 탈수와 파종성 감염을 일으킬 수 있으므로 입원과 항생제 치료가 필요할 수 있다[8].

Non-typhoidal *Samonella*는 다양한 동물의 위장관 내에서 집락을 형성하기 때문에 가금류 고기, 달걀, 다진 소고기, 우유, 야채, 과일 등 농수축산물이 오염되어 이를 섭취한 사람에게 전파되는 사례가 흔히 보고된다[11-18]. 또한 초콜릿 바, 땅콩 버터, 오렌지 주스, 훈제 연어 등 다양한 가공식품을 통한 전파 사례도 보고되고 있다[19-22].

부산시 10개 학교에서 동시에 발생한 장관감염증 집단발생 역학조사를 통해 원인병원체는 살모넬라 톰슨이며 감염원은 초콜릿 케이크로 확정할 수 있었다. 대부분의 학교에서 단봉형 유행곡선을 보여 단일 공동노출에 의한 유행임을 확인하였고 사례군의 증상이 설사와 발열을 주요 증상으로 하는 non-typhoidal *Salmonella*에 의한 위장관염과 일치하며 각 학교에서 급식을 섭취한 전 학년에서 사례가 고르게 분포하였다. 동일 회사에서 완제품 형태로 공급한 조각 초콜릿 케이크의 식품섭취 여부 오즈비가 5개 학교에서 공통적으로 의미있는 값을 보였고 그 중 2개 학교에서는 오즈비가 각각 39.4(95% CI 5.4-289.5), 30.3(95% CI 1.8-518.8)으로 매우 높았다. 10개 학교 모두 직장도말 검체 또는 초콜릿 케이크에서 살모넬라 톰슨이 검출되었고 PFGE 유형이 모두 동일하였다.

10개 학교 중 5개 학교는 9월 3일 중식, 나머지 5개 학교는 9월 4일 중식으로 초콜릿 케이크가 제공되었는데, 초콜릿 케이크가 제공된 날짜별 유행 곡선을 보면 대부분의 사례가 non-typhoidal *Salmonella*의 잠복기인 6-72시간 사이에 증상이 발생하였음을 확인할 수 있었다(Figure 2). 몇몇 학교의 경우 유행곡선의 꼬리가 길거나 사례 발생이 다시 증가하는 모양을 보이기도 하였는데, 이것은 단일 공동노출로 인한 사례 중 일부 긴 잠복기를 가지는 경우이거나 사람 간 전파로 인한 소규모 2차 유행으로 추정할 수 있다[9, 23].

F학교에서 보관 중이던 포장된 초콜릿 케이크는 양이 충분하여 크림만 따로 분리하여 검사가 가능하였는데 크림에서 살모넬라 톰슨이 검출되었다. 이후 식품의약품안 전처는 해당 초콜릿 케이크의 오염원으로 크림을 만들 때 사용된 난백액이 살모넬라 톰슨에 오염되었다고 발표하였다.

이 등은 우리나라에서 계란 또는 계란을 재료로 한 식품과 연관된 non-typhoidal salmonellosis의 집단발생이 보고되고 있음에도 달걀에서 살모넬라가 검출되었다는 연구 결과는 없음을 지적하면서 신뢰할 수 있는 자료를 생산할 수 있는 전국 조사가 강력히 요구된다고 하였다[24]. 식품매개 감염병 집단발생을 예방하기 위해서는 농수축산물 원재료부터 사람의 위장관염 원인병원체까지 통합적이고 체계적인 감시체계가 구축되어야 한다. 또한 지역에 국한되어 발생하는 식품매개 감염병 집단발생의 경우 식품의 원재료에 대한 역추적 조사가 거의 이루어지지 않고 있는데, 이번과 같은 전국적 대규모 집단발생을 예방하기 위해서는 식품의 원재료에 대한 역추적 조사가 보다 철저히 이루어져야 할 것이다.

부산에서 발생한 이번 대규모 유행은 10개 학교에서 동시에 발생하였는데 대부분 그 달에 생일을 맞는 학생들의 생일 축하를 위해 월초에 케이크가 제공되었기 때문이었다. 부산광역시 학교 중 9월 3일 월요일과 9월 4일 화요일에 해당 초콜릿 케이크가 급식으로 제공된 학교는 총 30개였고 이 중 유통기한이 2019년 4월 9일, 10일, 11일에 해당하는 초콜릿 케이크가 공급된 학교에서만 장관감염증이 집단발생하여 특정 날짜에 생산된 초콜릿 케이크가 오염되었을 것으로 추정할 수 있었다. 조리 없이 완제품 형태로 집단급식소에 제공되는 식품의 경우 생산 단계에서 오염되면 대규모 유행을 일으킬 수 있으므로 생산 단계에서의 안전관리가 더욱 강조되어야만 한다. 또한 동일한 완제품이 전국 학교에서 동시에 급식으로 제공된 것도 유행의 규모를 키운 주요 원인이었으므로 학교와 같은 큰 규모의 집단급식소에서 전국의 학생들이 동시에 동일한 식품에 노출되지 않도록 하는 조치가 필요하다.

이 조사의 제한점은 전교생을 대상으로 후향적 코호트 조사를 하지 못한 점과 사례군 1,111명 중 143명(12.8%)의 증상 발생 시간이 조사되지 않아 완전한 유행곡선을 그리지 못한 것이다. 전교생을 대상으로 후향적 코호트 조사를 하기 위해서는 하교 시간을 넘길 때까지 학생들을 남겨야 하는 부담이 있어 사례-대조군 연구로 조사를 진행할 수 밖에 없었다. 현재 사용되고 있는 종이 설문지가 아니라 온라인 설문법을 개발하여 조사 시간을 단축한다면 후향적 코호트 디자인의 조사를 적용하기 용이할 것이다. 3일 동안 10개 학교에서 동시에 집단발생이 보고되었기 때문에 완결성보다는 신속성에 초점을 맞추고 조사하였다. 설문조사에서 응답이 누락된 항목이 일부 있었으나 10개 학교에서 설문조사와 인체 검체, 보존식, 환경 검체 모두 누락 없이 시행할 수 있었던 것은 신속하게 조사가 이루어졌기 때문이다.

부산광역시에서 이러한 대규모 유행 역학조사가 신속하고 원활하게 이루어진 데에는 2017년 구성되어 꾸준히 운영되고 있는 ‘부산광역시 수인성 및 식품매개 감염병 실무 협의체’ 가 큰 역할을 하였다. 부산광역시 역학조사반, 보건환경연구원, 구·군 보건소, 식품위생부서, 교육청, 식품의약품안전청 등으로 구성된 협의체를 통해 학교 등에서 발생하는 감염병 집단발생 역학조사 진행을 논의하고 자료를 공유하는 체계가 이미 구축되어 있었기 때문이다. 이러한 실무 협의체와 같이 기관 간 혹은 부서 간 횡적이며 자발적인 협업 관계는 감염병 역학조사가 원활히 이루어지기 위해 매우 중요한 요소이다.

윤리 성명 Ethics Statement

The author has no conflicts of interest to declare for this study.

감사의 글 Acknowledgements

이 역학조사를 수행하고 자료 수집에 도움을 주신 부산광역시 금정구·부산진구·서구·영도구·해운대구 보건소와 각 학교 및 교육청 관계자 여러분께 감사드립니다.

참고 문헌 References

1. Korea Centers for Disease Control and Prevention. 2018 Guideline for Control of waterborne and foodborne disease. Cheongju: KCDC; 2018. p. 6-8 p. (Korean, author's translation).
2. Nam JG, Park GY, Sim SK, Lee YS LY. Infectious disease laboraory diagnosis; The test method of disease 3th ed. Seoul: National Institute of Health; 2005. p. 61-72 (Korean, author's translation).
3. Korean Food and Drug Administration (KFDA). Korean Food Code. General test methods: Test methods for proximate composition. Seoul: Korean Food and Drug Administration; 2018. p. 321-331 (Korean, author's translation).
4. Korea Centers for Disease Control and Prevention. PFGE(Pulsed Field Gel Electrophoresis) General test methods. KCDC. Osong: KCDC; 2008. p. 1-30 (Korean, author's translation).
5. Pagano M, Gauvreau K. Principles of biostatistics. Florida: CRC Press co; 2018. 342–373 p.
6. HALDANE BJ. The estimation and significance of the logarithm of a ratio of frequencies. Ann Hum Genet 1956;20(4):309–311.
7. Grimont, PA, Weill, FX. Antigenic formulae of the Salmonella servovars: WHO Collaborating Centre for Reference and Research on Salmonella. 9th ed, Institute Pasteur. Paris: WHO Institut Pasteur; 2007. p. 6-10
8. The Korean Society of Infectios Diseases(KSID). Infectious Disease. Seoul: koonja Co; 2014. p. 529-534 (Korean, author's translation).
9. Centers for Disease Control and Prevention. CDC Yellow Book 2018: Health Information for International Travelers. New York: Oxford University Press; 2017. p. 69-72
10. Heymann DL, editor. Control of Communicable Diseases Manual. 20th ed. Washington, DC: American Public Health Association; 2015. p. 532-533
11. Kimura AC, Reddy V, Marcus R, Cieslak PR, Mohle-Boetani JC, Kassenborg HD, et al. Chicken Consumption Is a Newly Identified Risk Factor for Sporadic Salmonella enterica Serotype Enteritidis Infections in the United States: A Case-Control Study in FoodNet sites. Clinical Infectious Diseases 2004;38(Supplement_3):S244–52.
12. Altekruse SF, Bauer N, Chanlongbutra A, DeSagun R, Naugle A, Schlosser W, et al. Salmonella enteritidis in broiler chickens United States 2000–2005. Emerging infectious diseases 2006;12(12):1848.
13. Centers for Disease Control and Prevention(CDC). Multistate outbreak of Salmonella typhimurium infections associated with eating ground beef--United States, 2004. Morbidity and mortality weekly report 2006;55(7):180.
14. Centers for Disease Control and Prevention(CDC). Salmonella typhimurium infection associated with raw milk and cheese consumption--Pennsylvania, 2007. Morbidity and

mortality weekly report 2007;56(44):1161.

15. Ryan CA, Nickels MK, Hargrett-Bean NT, Potter ME, Endo T, Mayer L, et al. Massive outbreak of antimicrobial-resistant salmonellosis traced to pasteurized milk. JAMA 1987;258(22):3269-74.
16. Centers for Disease Control and Prevention(CDC). Outbreaks of Salmonella infections associated with eating Roma tomatoes--United States and Canada, 2004. Morbidity and mortality weekly report 2005;54(13):325.
17. Centers for Disease Control and Prevention(CDC). Outbreak of Salmonella serotype Saintpaul infections associated with multiple raw produce items--United States, 2008. Morbidity and mortality weekly report 2008;57(34):929.
18. Mba-Jonas A, Culpepper W, Hill T, Cantu V, Loera J, Borders J, et al. A Multistate Outbreak of Human Salmonella Agona Infections Associated With Consumption of Fresh, Whole Papayas Imported From Mexico—United States, 2011. Clinical Infectious Diseases 2018;66(11):1756-61.
19. Gill O, Bartlett C, Sockett P, Vaile M, Rowe B, Gilbert R, et al. Outbreak of Salmonella napoli infection caused by contaminated chocolate bars. The Lancet 1983;321(8324):574-7.
20. Centers for Disease Control and Prevention(CDC). Multistate outbreak of Salmonella infections associated with peanut butter and peanut butter-containing products--United States, 2008-2009. Morbidity and mortality weekly report 2009;58(4):85.
21. Jain S, Bidol SA, Austin JL, Berl E, Elson F, Williams ML, et al. Multistate Outbreak of Salmonella Typhimurium and Saintpaul Infections Associated with Unpasteurized Orange Juice—United States, 2005. Clinical Infectious Diseases 2009;48(8):1065-71.
22. Friesema I, de Jong A, Hofhuis A, Heck M, van den Kerkhof H, de Jonge R, et al. Large outbreak of Salmonella Thompson related to smoked salmon in the Netherlands, August to December 2012. Eurosurveillance 2014;19(39):20918.
23. Blaser M. A review of human salmonellosis: I. Infective dose. Reviews of infectious diseases 1982;4(6):1096-106.
24. Lee S-K, Seo K-H. Egg Production Systems and Salmonella in Korea. Producing Safe Eggs. Seoul: Elsevier; 2016. p. 45-58
